# Neuropathologic features in the hippocampus and cerebellum of three older men with fragile X syndrome

**DOI:** 10.1186/2040-2392-2-2

**Published:** 2011-02-08

**Authors:** Claudia M Greco, Celestine S Navarro, Michael R Hunsaker, Izumi Maezawa, John F Shuler, Flora Tassone, Mary Delany, Jacky W Au, Robert F Berman, Lee-Way Jin, Cynthia Schumann, Paul J Hagerman, Randi J Hagerman

**Affiliations:** 1 MIND Institute, University of California-Davis Medical Center, Sacramento, CA, USA; 2 Department of Pediatrics, University of California-Davis Medical Center, Sacramento, CA, USA; 3 Department of Pathology and Laboratory Medicine, University of California-Davis, School of Medicine, Sacramento, CA, USA; 4 Graduate Program in Neuroscience, University of California-Davis, Davis, CA, USA; 5 Department of Neurological Surgery, University of California-Davis, Davis, CA, USA; 6 NeuroTherapeutic Research Institute, University of California-Davis, Davis, CA, USA; 7 Department of Biochemistry and Molecular Medicine, University of California, School of Medicine, Davis, California, USA; 8 Elwyn Fragile X Center, Elwyn, PA, USA; 9 Department of Psychiatry and Behavioral Sciences, University of California, School of Medicine, Davis, California, USA

## Abstract

**Background:**

Fragile X syndrome (FXS) is the most common inherited form of intellectual disability, and is the most common single-gene disorder known to be associated with autism. Despite recent advances in functional neuroimaging and our understanding of the molecular pathogenesis, only limited neuropathologic information on FXS is available.

**Methods:**

Neuropathologic examinations were performed on post-mortem brain tissue from three older men (aged 57, 64 and 78 years) who had received a clinical or genetic diagnosis of FXS. In each case, physical and cognitive features were typical of FXS, and one man was also diagnosed with autism. Guided by reports of clinical and neuroimaging abnormalities of the limbic system and cerebellum of individuals with FXS, the current analysis focused on neuropathologic features present in the hippocampus and the cerebellar vermis.

**Results:**

Histologic and immunologic staining revealed abnormalities in both the hippocampus and cerebellar vermis. Focal thickening of hippocampal CA1 and irregularities in the appearance of the dentate gyrus were identified. All lobules of the cerebellar vermis and the lateral cortex of the posterior lobe of the cerebellum had decreased numbers of Purkinje cells, which were occasionally misplaced, and often lacked proper orientation. There were mild, albeit excessive, undulations of the internal granular cell layer, with patchy foliar white matter axonal and astrocytic abnormalities. Quantitative analysis documented panfoliar atrophy of both the anterior and posterior lobes of the vermis, with preferential atrophy of the posterior lobule (VI to VII) compared with age-matched normal controls.

**Conclusions:**

Significant morphologic changes in the hippocampus and cerebellum in three adult men with FXS were identified. This pattern of pathologic features supports the idea that primary defects in neuronal migration, neurogenesis and aging may underlie the neuropathology reported in FXS.

## Background

Fragile X syndrome (FXS) is the most common inherited form of intellectual disability, and the fragile X mental retardation 1 (*FMR1*) gene is the most common single-gene mutation associated with autism [1-5]. Approximately 30% of individuals with FXS meet all criteria of the *Diagnostic and Statistical Manual of Mental Disorders, Fourth Version *(DSM IV) criteria for autism as assessed with standardized measures (Autism Diagnostic Observation Schedule (ADOS) and Autism Diagnostic Interview, Revised: ADI-R (ADI-R)), and an additional 30% of those with FXS have PDD NOS (pervasive developmental disorder, not otherwise specified) [6].

The *FMR1 *protein (FMRP) is absent or deficient in individuals with FXS. FMRP is an RNA-binding protein that regulates the translation of a number of mRNAs whose protein products are important for synaptic development, maintenance and plasticity. In the absence of FMRP, production of many synaptic proteins are dysregulated, such as postsynaptic density protein (PSD)95, Arc (Arg 3.1), matrix metalloproteinase (MMP)9, the α-amino-3-hydroxyl-5-methyl-4-isoxazole-propionate (AMPA) receptor subunits, glutamate receptor (GluR)1 and GluR2, and Ca^2+^/calmodulin-dependent protein kinase (CaMK)II [7-9]. Other proteins regulated by FMRP are involved with axon guidance and cell motility, including microtubule-associated protein (MAP)1B and Semaphorin 3F, suggesting that dysregulation of these proteins in the absence of FMRP may be related to the periventricular heterotopia previously reported in some cases of FXS [10-13]. Furthermore, FMRP interacts with several proteins that have been identified as potential candidate genes underlying a number of neurodevelopmental disorders, such as the neuroligin family, neurorexin 1, SH3 and multiple ankyrin repeat domains (SHANK 3), phosphatase and tensin homolog (PTEN), mammalian target of rapamycin (mTOR), PSD95, Wnt7a and Arc (Arg 3.1) [7,14-20]. A recent report by Luo *et al. *[20] demonstrated that FMRP in the mouse regulates the protein expression of several components crucial for adult neurogenesis, including cyclin-dependent kinase (CDK)4, cyclin D1 and glycogen synthase kinase (GSK)3β. Dysregulation of GSK3β leads to reduced Wnt signaling pathway activity, which alters the expression of neurogenin 1 and the fate specification of adult neuroprogenitor cells.

Despite recent advances in our understanding of the molecular pathology underlying FXS, only limited neuropathologic information is available (Table 1). To address this issue, we analyzed the hippocampus and cerebellum in three men with FXS using histologic, immunochemical and molecular techniques. Neuropathologic abnormalities were evident in both the hippocampus and the cerebellum of all three cases. The CA1 region of the hippocampus showed local thickening and abnormal undulations, and appeared to be enlarged relative to age-matched, non-FXS control hippocampi. The cerebellum, particularly lobules VI to VII of the vermis, was reduced in size and displayed reduced Purkinje cell (PC) density compared with age-matched, non-FXS controls. These findings correspond to previously reported studies of individuals with FXS using magnetic resonance imaging (MRI), in which dysmorphic, enlarged hippocampi, decreased cerebellar size, and preferential atrophy of vermal lobules VI to VII were observed [21-23].

**Table 1:** Documented neuropathology in previously published studies

Author	Patient	Tissues	Brain region analyzed	Method of analysis	Microscopical neuropathology
Dunn *et al*, 1963 [12]	18-year-old man, later diagnosed with fragile X syndrome	Brain: 1040 g,^1 ^normal cortical pattern, mild ventricular dilatation	Multiple regions	LM^2^	Inc neurons in subcortical white matter; reduced myelin in cerebral white matter; siderosis of globus pallidus, inferior olivary heterotopia,

Rudelli *et al*, 1983 [59]	23-, 24-week fetuses	Brains(2) showing normal cortical development; testes		Gross examination only	None noted

Rudelli *et al*, 1985 [60]	62-year-old male	Brain: mild cortical atrophy	Parieto-occipital neocortex	LM, Golgi, EM^3^	Increased long, thin, immature spines; decreased synaptic length by EM

Desai *et al*, 1990 [13]	33-year-old male with ALS^4^	Brain (1850 g) with ALS pathology; testes	Whole brain	LM	Heterotopia of olivary nucleus; Subcortical white matter neuronal clusters

Hinton *et al*, 1991 [61]	15-, 41-, 62-year-old male patients (62-year-old in Rudelli, 1985 [60])	Brains (3):normal	Parieto-occipital neocortex	Golgi	Increased long, thin spines
			
			Cingulate, temporal association cortex	Morphometric analysis	No significant differences in neuronal counts

Wisniewski, 1991 [62]	63-year-old man	Brain: mild atrophy, hydrocephalus, AVM^5 ^of left temporal lobe	Unspecified neocortex	Golgi	Increased long, thin spines

Sabaratnam, 2000 [63]	67-, 87-year-old men	67-year old (1778 g). 87-year-old: brain enlarged, ventricular dilatation	87-year-old: hippocampus, cerebellum	LM	CA4 cell loss, gliosis; PC^6 ^dropout, Bergmann gliosis

Irwin *et al*, 2001[64]	48-, 48-, 73-year-old men	Brain	Temporal and visual neocortex	Golgi	Increased long, thin spines; increase in spine density

Moro *et al*, 2006 [11]	4.5-year-old and 13-year-old boys	Live patients		MRI^7^	Periventricular heterotopias in both cases

^1^Brain weights within normal limits unless otherwise noted.

^2^Light microscopy, standard histologic stains.

^3^Electron microscopy.

^4^Amyotrophic lateral sclerosis.

^5^Arteriovenous malformation

^6^Purkinje cell.

^7^Magnetic resonance imaging.

## Case histories

### Patient 1

This man was the product of a full-term pregnancy. He had global developmental delay, and was admitted to a residential facility at 15 years of age, because of difficulties with aggression. He was diagnosed with FXS aged 52 years; the findings of the physical examination included large prominent ears, a long narrow face and macroorchidism. At this time, he had a full-scale intelligence quotient (FSIQ) of 34, which had fallen to 22 at the age of 65 years. A chromosomal analysis at 52 years of age yielded 23% fragile X-positive metaphases.

The patient was a social individual, who befriended his caretakers and peers. He repeated words and phrases, and required no psychotropic medications. Over time, he developed high blood pressure, prostate cancer and osteoporosis, and a seizure disorder at 75 years that required treatment with phenytoin. At the age of 77 years, he was diagnosed with gait ataxia and began treatment with amantadine, without therapeutic benefit. Computed tomography (CT) of the brain revealed cerebral and cerebellar atrophy and ischemic white-matter changes. Within the last six months of his life, the patient was diagnosed with dementia, chronic obstructive pulmonary disease and a left bundle branch block. At 78 years, he presented to the hospital with abdominal pain and 'coffee-ground' emesis, and died within 24 hours of admission. Genomic analysis of DNA isolated from post-mortem brain tissue established his full mutation status, with hypermethylated, full mutation alleles (CGG repeats of 339, 486, 619, 755, 938, 1225).

### Patient 2

This man was the product of a normal pregnancy. He had developmental delay in early childhood, and had severe language deficits; he was never able to speak in full sentences. He was hyperactive and anxious throughout childhood, and showed excessive rocking, hand-flapping, poor eye contact, tactile defensiveness and destructive tantrums, resulting in a diagnosis of autism. He entered a group home at 30 years of age, where he required antipsychotic medications for behavior problems.

On physical examination, he was found to have mitral valve prolapse. The family history included a brother with FXS, and a sister who was a fragile X premutation carrier and mother of two daughters with FXS. The patient's mother had had severe depression in her 40s, developed unsteady writing and dementia in her 80s, and was still alive at 94 years at the time of this patient's death. The patient died at the age of 57 years despite resuscitative efforts after choking on food. Molecular testing on postmortem brain tissue demonstrated the presence of a full mutation allele (436 CGG repeats).

### Patient 3

This man was born after a normal pregnancy, and had delayed development. His physical features included a long face, prominent ears and macroorchidism. Abnormal behavioral features included anxiety and hand-biting. The patient was shy but interactive, and he was able to speak in full sentences. He had savant skills for trivia, and he had an FSIQ of 57 in adulthood. He lived in a group home, and was productively employed.

In his fifties, the patient showed increased anxiety and increased self-abusive behaviors, and he became increasingly rigid in his routines. He developed hypothyroidism that required levothyroxine, and hyperlipidemia that was treated with simvastatin. At the age of 64 years, the patient developed pain in his abdomen, was diagnosed with a primary liver neoplasm, and died within 3 months without a definitive tumor-tissue diagnosis. Southern blot analysis of DNA isolated from post-mortem brain tissue showed methylation mosaicism, with a hypermethylated, full mutation allele (429 CGG repeats) present in approximately 72% of the cells, with the remaining alleles unmethylated, and ranging from approximately 340 to 440 CGG repeats.

## Methods

### Pathology

#### Autopsies

Brain autopsies were performed in accordance with protocols approved by the University of California-Davis institutional review board and informed consent was obtained from family members.

One hemisphere of the fresh brain was cut into coronal blocks 10 mm thick, and frozen at -80°C; the remaining hemisphere was fixed in 10% phosphate-buffered formalin. The temporal lobe was separated from the fixed hemisphere, and serially sectioned into blocks 5 mm thick for serial embedding of the amygdaloid-hippocampal complex. The blocks were embedded in paraffin wax and cut into serial sections 5 μm thick. The brainstem was separated from the cerebrum at the level of the midbrain. The cerebrum was coronally sectioned, the brainstem was sectioned in the perpendicular plane, and the cerebellum was cut into sagittal blocks 5 mm thick. The blocks were embedded in paraffin wax and cut into serial sections 5 μm thick. Brain sections were inspected grossly for abnormalities. Sampling for paraffin-wax embedding was performed for each case as follows: serial sections of the hippocampal complex, midline samples of each of the three vermal lobules, and one section of the lateral posterior lobe of the cerebellar cortex.

Matched samples were taken from five neurologically normal controls matched for age and gender (mean ± SD, 70 ± 9 years, range 60-8) obtained from the autopsy tissue repository at the University of California, Davis Medical Center Department of Pathology. Fixation, sampling, processing and staining were equivalent for control and FXS brains. Post-mortem intervals (PMIs) were, on average, considerably shorter for the FXS brains than for controls (FXS 16.3 ± 4.4 hours, range 11.5-20 hours; controls 52.0 ± 43.6 hours, range 12.5-119 hours).

For the purposes of this study, the vermal lobules of the cerebellum are referenced as follows: the superior lobule (lobules I to V of the anterior lobe of the cerebellum), and the posterior (lobules VI to VII) and inferior lobule (lobules VIII to X) of the posterior lobe of the cerebellum.

#### Histology and immunochemistry

All tissue blocks were processed for paraffin-wax embedding, histochemical staining and immunohistochemistry using previously reported techniques [24]. All histologic, immunohistochemical and counting techniques were performed on 5 μm thick wax-embedded sections, as previously reported [24]. Briefly, tissue blocks were processed for paraffin-wax embedding using standard techniques. Staining was performed according to standard methods, using hematoxylin and eosin (H&E) and Luxol fast blue (LFB) counterstained with periodic-acid-Schiff (PAS) (for myelin). Silver (modified Bielschowsky) stain for axons was then used on selected cases [24]. Immunohistochemical staining using anti-ubiquitin, anti-glial fibrillary acidic protein (anti-GFAP), anti-CD68 (KP-1; Dako, Carpenteria, CA, USA), anti- leucocyte common antigen (LCA/CD-45; DakoCytomation, Glostrup Denmark) and anti-neurofilament (NF) (Dako) antibodies was performed using methods reported previously [24]. Dual-label (myelin basic protein (MBP)/ubiquitin) immunohistochemistry was performed using the avidin-biotin procedure, with anti-MBP antibody (SM 194; Sternberger Monoclonals, Baltimore, MD, USA) visualized with diaminobenzidine and anti-ubiquitin antibody (Dako, Glostrup, Denmark) visualized with Nova Red (Vector Laboratories, Burlingame, CA, USA). Appropriate positive and negative controls were employed for each antibody.

#### Immunofluorescent staining for calbindin

Wax-embedded sections 5 μm thick were dewaxed and blocked in 10% goat serum (Zymed, San Francisco, CA, USA) with 0.5% Triton X-100 for 1 hour, followed by 1% non-fat dry milk with 0.1% Triton X-100 for 30 minutes at room temperature. Sections were then incubated with 1:150 anti-calbindin antibody (Chemicon, Billerica, MA USA) diluted in 1% bovine serum albumin with 0.1% Triton X-100 at 4°C for 48 hours, followed by the secondary antibody, 1:600 rabbit anti-IgG conjugated with Alexa 568 (Molecular Probes, Carlsbad, CA USA) for 1 hour at room temperature. Sections were then counterstained with 4',6-diamidino-2-phenylindole (Vector Laboratories), observed under a microscope (Eclipse E600, Nikon, Melville, NY, USA) and digitally photographed (SPOT RTke; SPOT Diagnostics, Sterling Heights, MI USA).

#### Estimation of PC density in the cerebellum

Owing to the advanced age of the three patients with FXS (57, 64 and 78 years) included in the present study, and to reports of reductions in PC number with normative aging, counts of the number of PCs in the three cases of FXS were compared with corresponding counts in the five age-matched non-FXS cerebella. The areas evaluated for PC number were from the superior, posterior and inferior vermal lobules, and one section of the lateral posterior lobe cerebellar cortex, stained for H&E. In all cases, the portions of the lobules to be analyzed were marked on the slide before any microscopic analysis to reduce bias in selection of regions of interest (ROI), and the experimenter was blinded to the origin of the tissue sample (that is, which lobule was on which slide).

PCs were counted within defined ROI in the cerebellum using computer software to outline the ROI and then randomly select PCs for counting within the ROI (StereoInvestigator, version 8.0; Microbrightfield Inc., Williston, VT, USA). Briefly, at 4× magnification the PC layer (PCL) was outlined, taking care to not include the molecular or internal granular cell (IGL) layers. Tracing of ROI was completed for each of three 5 μm thick serial sections, separated by 25 μm. The program was then used to cast a regularly spaced counting grid over the traced ROI, and individual 25 × 25 μm counting frames were defined within each grid. PCs within each of 100 counting frames were then counted at 400× magnification (E-600 microscope, Nikon). PCs were identified morphologically, and PC nuclei rather than nucleoli were counted, as nucleoli in the PC do not stain reliably [25]. Any PC nuclei falling within or contacting the inclusion zones of each counting frame were counted, whereas any nuclei contacting the exclusion zones were not counted. After all counting frames were evaluated, the linear length (in mm) of the PC layer was measured and recorded. The number of PCs in the PCL and the linear length of the PCL were used to calculate the number of PC per mm as described previously [25].

The limited quantity of tissue available (three sections as opposed to a full serial set through the cerebellum) prevented a stereologic estimate of total PC numbers. Therefore, data are presented as the mean number of PCs counted per millimeter length of the PCL (that is, a density measurement [25]). In this way, we strove to be as conservative as possible to ensure that any reduction in PC number in FXS found would be underestimated rather than overestimated.

Additionally, as there have been reports of decreased cerebellar size in FXS [21], foliar cross-sectional width was measured at 20× magnification using the 'quick measure' line function on the StereoInvestigator software. These measurements were routinely taken at the midpoint of the folia to standardize the relative location of the measurements. Each measurement was taken thee times, and the average of the three measurements is reported. Additionally, each section was evaluated twice on separate days using the same procedures, and similar results were obtained.

#### Molecular measures

Genomic DNA was isolated from 500 mg of frozen cortex and cerebellum from the three patients with FXS using standard methods (Qiagen, Valencia, CA, USA). In two cases (patients 2 and 3), DNA was also extracted from whole blood (3 to 5 ml); results (CGG repeat sizes) were consistent with those obtained in brain tissue. CGG repeat size and methylation status were determined using both PCR and Southern Blot analysis using an image detection system (FluorChem 8800; Alpha Innotech, San Diego, CA, USA) as previously described [26]. For Southern blot analysis, 10 μg of DNA were digested with *Eco*RI and *Nru*I, and the Stb12.3 *FMR1 *genomic sequence, labeled with Dig-11-dUTP, was used as a probe. Genomic DNA was also amplified by PCR using the 'c' and 'f' primers described by Tassone *et al. *[26].

## Results

### Pathology

#### Gross examination

The cerebrums of patients 1 and 3 were grossly normal, with mild cortical atrophy deemed appropriate for age. Patient 2 had moderate ventricular dilatation, and prominent atrophy of the hippocampus and amygdala. The cerebella of all three cases appeared generally decreased in size. On midline sagittal sectioning through the vermis, both the superior and posterior lobar divisions were atrophied compared with age-matched control brains. Of the three patients, only patient 2 showed preferential atrophy of the posterior lobule by visual inspection. The inferior lobule appeared the least atrophied of the vermal lobules in all three patients. Sagittal sections of the lateral posterior lobe cortex from all patients showed no obvious atrophy upon visual inspection.

#### Microscopic examination

##### Hippocampus

**Figure 1:**
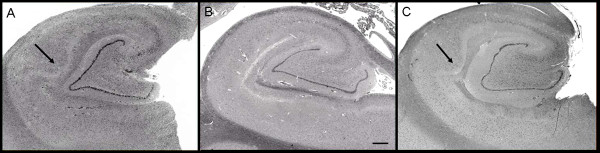
**Hippocampal formation**. Images of the hippocampal formation at the level of the lateral geniculate body from (A) patient 1 and (C) patient 3 show abnormal expansion of CA1 by increased numbers of pyramidal neurons. These are compared with the more usual hippocampal microarchitecture that shows a thinner linear band of neurons in CA1, as seen in (B) a 62-year-old male control. Haematoxylin and eosin, original magnification ×10; scale bar = 1 mm. Arrow indicates bulge/expansion composed of increased numbers of pyramidal cells in (A) patient 1 and (C) patient 3.

Because the structural features of the CA1/Sommer sector of the hippocampus were overshadowed by ischemic changes in patient 2, we limited our analysis of hippocampal microscopic structure to patients 1 and 3. In both cases, in several sections throughout the hippocampus evaluated, CA1 had similar abnormalities in the microanatomic organization of the pyramidal cell layer. There was a bulge or expansion composed of increased numbers of pyramidal cells (Figure 1) arranged in an undulating pattern. In neighboring regions, there appeared to be a reduction in pyramidal cell number. In all three cases, the penetrating cortical arterioles of the temporal cortex showed vascular hyalinosis. These vessel walls were negative for amyloid birefringence with Congo red stain, and showed faint staining with ß-amyloid antibody (not shown). Immunostaining was negative in brain parenchyma for β-amyloid, α-synuclein, ubiquitin and tau, stains that are routinely used for the neuropathologic diagnosis of the most commonly occurring neurodegenerative disorders, such as Alzheimer's disease, Lewy Body disease and frontotemporal dementia.

##### Cerebellum

**Figure 2:**
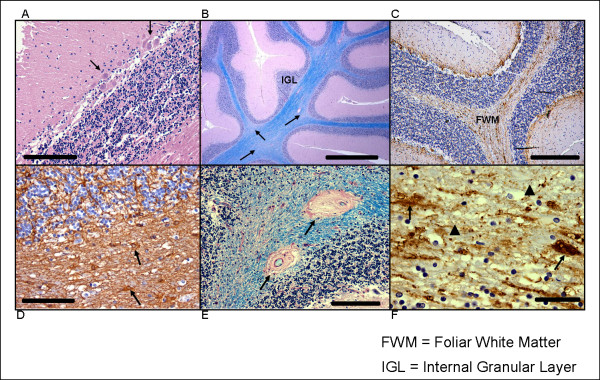
**Abnormalities in the cerebellum**. Histologic abnormalities seen in FXS cerebella. (A) Decreased numbers of Purkinje cells (PCs) and clusters of PCs (arrows) in which cells are often misoriented. Haematoxylin and eosin, original magnification ×200; scale bar = 200 μm. (B) Patchy pallor of foliar white matter and increased variability in thickness of the internal granular cell layer (IGL). Arrows indicate foci of myelin pallor. Luxol fast blue-periodic-acid-Schiff (LFB-PAS), original magnification ×200; scale bar = 1 mm. (C) Diffuse axonal loss in cerebellar foliar white matter (FWM) (neurofilament immunohistochemistry, original magnification ×100; scale bar = 400 μm); (D) Activated astrocytes in foliar white matter, a finding not seen in deep cerebellar white matter. Arrows indicate abnormal astrocytes. (glial fibrillary acidic protein immunohistochemistry, original magnification ×200; scale bar = 200 μm). (E) Vascular hyalinosis within foliar white matter, as indicated by arrows. LFB-PAS, original magnification ×200; scale bar = 200 μm. (F) High magnification of foliar white matter axonal abnormalities that include axonal loss (arrowheads) and swollen axons, as indicated by arrows. Neurofilament immunohistochemistry, original magnification ×400; scale bar = 100 μm.

Histopathologic abnormalities identified by H&E stain in the vermal lobules and the lateral posterior cerebellar cortex were similar in all three patients. Prominent, patchy loss of PC could be visualized with either H&E or calbindin stains. Quantitative analyses revealed a decrease of more than 40% in the number of PC (Figure 2) in all vermal lobules and lateral cortical sections compared with age-matched controls. Existing PCs were frequently clustered, and within these small groups, often lacked proper orientation. Compared with age-matched normal controls, there were increased numbers of PC in the IGL. Bergmann glia were present in expected numbers, and showed no increase in cytoplasmic staining for GFAP, contrary to what would be expected in Bergmann gliosis. There were small numbers of scattered GFAP-positive astrocytes in folia that demonstrated mildly expanded cytoplasmic borders; however, these changes fell short of those seen with either reactive or gemistocytic astrocytic morphology. Vellate astrocytes of the IGL showed no changes on GFAP immunostaining. Myelin staining (LFB-PAS) showed mild patchy pallor, most often in distal foliar white matter. NF staining revealed foci of scattered, swollen and fractured axons that corresponded to regions of myelin pallor. Within the distal foliar white matter, there were scattered white-matter small vessels with prominent hyalinosis of their walls, which were not seen in controls.

**Figure 3:**
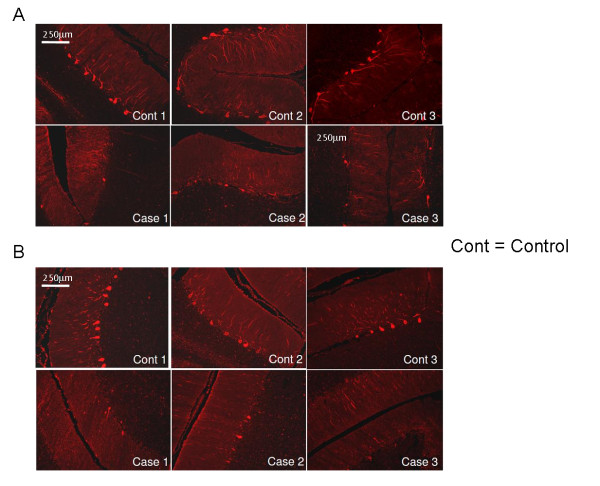
**Calbindin immunofluorescent staining in the cerebellar cortex**. Calbindin immunofluorescent staining of the cerebellar cortex indicates decreased numbers of PCs and diminished complexity of the PC dendritic arborization compared with age-matched controls. Similar changes were observed in all vermal lobules and in lateral posterior lobe cortex. (A) Vermal inferior lobule and (B) lateral posterior lobe cerebellar cortex. Original magnification ×10; scale bar = 250 μm.

Based on the improved characteristics of calbindin antibody staining over standard Nissl stains as a marker for PCs in postmortem human brains [25], we used calbindin immunofluorescence (IF) to evaluate the morphology of PCs and to confirm any reductions in cell number identified in H&E-stained cerebella. In age-matched controls (Figure 3), calbindin-immunoreactive PCs were spaced regularly, although somewhat variably, depending on the plane of the section, and the dendrites of control PCs had fine processes from their dendritic trees. By contrast, the three patients had a substantial reduction in calbindin-immunoreactive PCs, with attenuation of dendritic arborization. Similar features were seen throughout the vermis and lateral cortex.

#### PC density in the cerebellum

**Figure 4:**
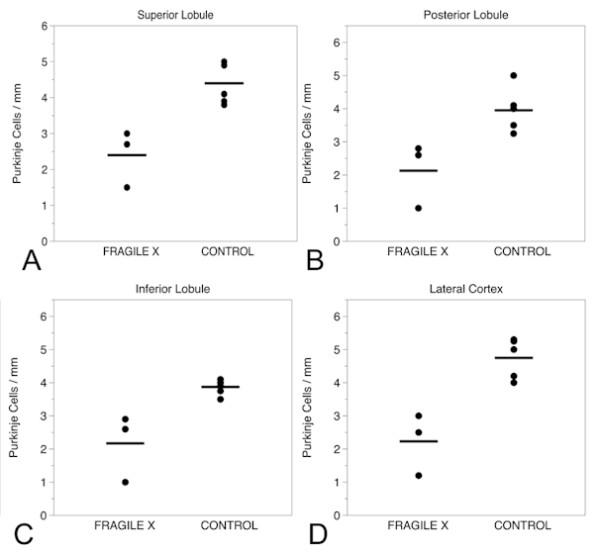
**Purkinje cell counts in the cerebellum**. Purkinje cell (PC) counts in: (A) superior lobule; (B) posterior lobule; (C) inferior lobule; and (D) lateral cortex, in the three fragile X cases and age-matched controls.

Based on the cell-density measurements performed in the present study, there was a 52% reduction in PCs per mm in the superior lobule, a 54% reduction in the posterior lobule, a 49% reduction in the inferior lobule and a 58% reduction in the lateral cortex sample, compared with control cerebellar sections. There was no numerical overlap between counts of PCs between the cases and controls; all patients with FXS had clear reductions in PC density in all cerebellar regions evaluated (Figure 4A-D). These results suggest a global reduction in PC number in the patients with FXS relative to the age-matched non-FXS controls. This reduction in PC number was supported by the calbindin IF results.

**Figure 5:**
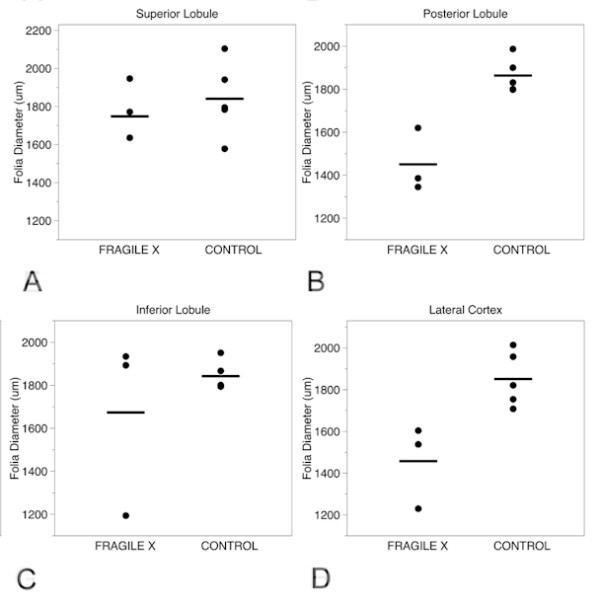
**Foliar diameter in the cerebellum**. Foliar width measurements in: (A) superior lobule; (B) posterior lobule; (C) inferior lobule; and (D) lateral cortex, in the three fragile X cases and age-matched controls.

To further study possible microscopic correlates to previously observed MRI abnormalities in the cerebellar vermis in FXS, we obtained simple measurements of foliar width. There were no dramatic reductions in foliar widths in the superior and inferior vermal lobules (0% and 7% reduction, respectively, relative to controls); visual inspection of this data revealed a large overlap in foliar widths between cases with FXS and controls (Figure 5A-D). However, there was a dramatic reduction in foliar width in the posterior lobules (21% reduction) and in the lateral cortex of the cerebellum (20% reduction) compared with controls. Foliar width measurements of the inferior lobules gave similar values to controls for patients 1 and 3. Patient 2 also had reduced foliar width in the inferior lobule, (Figure 5C). There was no numerical overlap between patients with FXS and controls; all cases with FXS had clearly reduced foliar widths in the lateral cortex and the posterior lobule compared with all of the controls evaluated. These data suggest that lobules VI to VII are decreased in size in FXS compared with non-FXS controls, but that lobules I to V and VIII to X appear to be the same size as non-FXS controls, consistent with published MRI findings, which identified a similar pattern.

## Discussion

We have identified novel microscopic abnormalities in the hippocampal formation and cerebellar vermis in three aged men with FXS. These offer correlations with established MRI changes in these structures in individuals with FXS. Neuroimaging studies of FXS have shown enlargement of the hippocampi [21,22,27,28] and decreased cerebellar size, particularly the posterior lobe of the cerebellum. In patients 1 and 3, the CA1 pyramidal cell layer of the hippocampus was focally enlarged and widened (Figure 1). Such microscopic malformations of the hippocampus have rarely been identified in the medical literature. 'Tectonic hippocampal malformation', described to date only in temporal lobe epilepsy [29], appear similar to the hippocampal pathology we found. It is likely that this finding in FXS may be related to abnormalities in adult neurogenesis, as FMRP deficiency has been reported to increase the early proliferation of both stem and progenitor cells in addition to reducing the survival rate of young neurons [20]. In addition, the loss of FMRP down-regulates the Wnt pathway, which is important for cell proliferation in the hippocampus [20]. Therefore, focal proliferation adjacent to focal cell loss, representing a focal dysplasia, may be related to the dysregulation of a number of proteins in the absence of FMRP. The dysregulation of the Wnt pathway in the absence of FMRP also relates to dysregulated neural migration that appears to underlie some neuropathologic features of FXS [11].

The first neuroanatomical abnormality in FXS visualized by MRI was the decreased size of the posterior lobe of the cerebellar vermis [30]. Using MRI, Gothelf *et al. *[23] studied 84 children with FXS who ranged in age from 1.1 to 22.7 years (range 11.7 ± 5.7) and 72 age-matched controls, and found that size of the posterior lobe of the vermis (lobules VI to X) correlated positively with FSIQ and FMRP levels. Particular atrophy of lobules VI to VII of the vermis on MRI has also been described [23,30]. Our measurements of foliar width support these previous studies by demonstrating decreased width of the folia in the posterior lobule compared with the adjacent superior and inferior lobules. We have also identified a 40% reduction in PCs in the vermis and in a limited analysis of the cortex of the lateral posterior lobe. In all areas of cerebellum examined, there was disorientation and misplacement of PC, mild conformational changes in the microscopic appearance of the IGL, and axonal and myelin pathology in the foliar white matter.

Decreased cerebellar vermis size is a common finding in other neurodevelopmental disorders, including attention deficit hyperactivity disorder (ADHD), autism, 22q11.2 deletion syndrome (velocardiofacial syndrome), Joubert syndrome and schizophrenia [31-36]. Additionally, PC count is reduced in some cases of autism [25,36-38], and PC size is reduced in schizophrenia [39,40]. In addition to a reported negative correlation between vermis volume and autistic tendencies in both autism and FXS [38,41], it is suggested that the vermis may modulate emotion in schizophrenia, and underlie some of the pathogenesis behind the disease [39]. Hessl *et al. *[27] suggested that a subset of behavioral irregularities seen in FXS, such as perseverative speech, hyperactivity, tactile defensiveness, language dysfunction and attention deficits, could be related to improper functioning of the cerebellar vermis.

Studies on schizophrenia and autism have also revealed features in the hippocampal formation and cerebellum consistent with migration disorders. Heterotopic displacement and aberrantly clustered neurons, particularly pre-alpha cells, in laminae II and III have been reported in the schizophrenic entorhinal cortex [42-44]. Owing to the interconnections of the entorhinal cortex and hippocampus, and its connections to other cortical areas, such migration abnormalities could contribute significantly to the neuropsychologic deficits in schizophrenia [42]. Cases of autism have been reported in which heterotopic cells were observed in the cerebellar molecular layer [37], in the white matter of the anterior cingulate gyrus and inferior frontal gyrus, in the hippocampus, the wall of the lateral ventricle [45] and lateral to the olivary bodies [46]. However, these findings in autism are limited because of a dearth of available tissue. In addition, conflicting evidence has arisen from other studies attempting to replicate the findings in schizophrenia [47-49]. It is interesting that a recent report of FMRP levels in neuropsychiatric disorders without an *FMR1 *mutation demonstrated very low levels of FMRP in the brains of people with schizophrenia [50] and people with autism [51]. Therefore, low levels of FMRP in both autism and schizophrenia would be likely to lead to an overlap or commonality of neuropathologic findings between these disorders and FXS.

The *FMR1 *gene has been implicated in neuronal migration anomalies [11]. Specifically, there have been previous observations of periventricular heterotopia in neuropathologic studies of FXS [12,13], and on magnetic resonance imaging (MRI) scans in two cases of FXS [11]. The absence of FMRP has been reported to dysregulate molecules involved in axon guidance and mobility, including Wnt 7a, semaphorin 3F and MAP1B, possibly leading to migration abnormalities similar to those we report here [10,11,17,20]. A recent study of the cytoplasmic *FMR1 *interacting protein (CYFIP1) bacterial artificial chromosome (BAC) transgenic mouse, which over-expresses CYFIP, demonstrated similar disorientation and misalignment of the PCs in addition to abnormalities of the dendritic tree detected by Golgi staining, similar to our findings with calbindin staining [52]. CYFIP is regulated by FMRP, and it is an interacting protein with FMRP that is crucial to the repression of proteins important for synaptic plasticity by FMRP [53]. Expression of CYFIP is dysregulated in FXS [54]. Further investigation is therefore warranted to elucidate prenatal or perinatal migratory mechanisms involved in FXS, autism and other neurodevelopmental disorders.

Our findings provide insight into the structural correlates that relate to functional deficits involving the cerebellum in FXS. The decreased density of PCs, which constitute the primary efferents from the cerebellar cortex, in these older men may be related to (or result from) abnormal GABAergic transmission and disrupted cortical-cerebellar connectivity [55,56]. The cerebellum is the site of termination of the spinocerebellar pathway, which carries subconscious proprioception [27]; and is thus involved in motor functions including the relation of external responses to a person's own acts, and the ability to expect the outcomes of particular movements, which are impaired in FXS [27]. A recent study of the medical and neurologic problems of aging in FXS in over 60 individuals demonstrated a high rate of motor problems including parkinsonian features of tremor and ataxia [57]. In addition, the recent report of the role of FMRP in neurogenesis has shown deficits in neurogenesis in cells with an absence of FMRP [20]. These findings may have significant effects on the aging process of those with FXS and should stimulate further neuropathologic studies in FXS.

## Conclusion

Our neuropathologic analysis of post-mortem CNS tissue from three patients with FXS provides clear evidence of cytoarchitectural abnormalities of the aging brain in individuals with FXS. We speculate that these abnormalities may reflect defects in neuronal migration and neurogenesis, and neuronal function and maintenance in aging individuals [58]. Our results also reveal microscopic pathologic features in FXS brains that overlap with those reported in cases of autism and schizophrenia. These similarities suggest that shared pathogenic mechanisms may underlie these developmental disorders, and the recent reports of low FMRP in the brain of people who have died from schizophrenia or autism further supports this concept [50,51]. Additional neuropathologic studies may also identify a degenerative component that corresponds to the clinical neurologic deterioration often seen in individuals with FXS as they age [57]. The results from the current study warrant more comprehensive studies on a larger population of cases with FXS, which requires a coordinated effort in collecting and studying these brain samples. Our hope is that correlation of this information with molecular and clinical studies will increase our understanding of this and other genetic disorders, and result in early treatment that may diminish or nullify the developmental and aging effects of this genetic disorder.

## Competing interests

RJH has received funding from Seaside Therapeutics, Roche, Forest, Johnson and Johnson, Curemark, Novartis and Neuropharm related to treatment studies in individuals with fragile X syndrome, FXTAS and/or autism. PJH and FT have submitted a patent application for a fragile X screening method. The authors have no financial conflicts.

## Authors' contributions

CMG designed this study. CMG and LWJ performed the neuropathologic analysis of the brain tissues, and both participated in writing the manuscript. RFB and MRH designed the quantitative analyses of PC density, performed the quantitative measurements of PC number, and helped draft the manuscript. MRH confirmed the hippocampal pathologic findings, and designed and performed analyses of foliar width, blinded to patient history. LWJ performed and analyzed the calbindin IF analysis of the cerebellum. FT performed molecular analyses. RJH, JWA, MD and JS gathered clinical information and helped to write the manuscript. LWJ, PJH, RFB and RJH provided funding support, participated in study design and coordination, and helped to draft and revise the manuscript. CS helped to revise the manuscript. All authors read and approved the final manuscript.
